# Risk of HCC decreases in HBV-related patients with cirrhosis acquired recompensation: A retrospective study based on Baveno VII criteria

**DOI:** 10.1097/HC9.0000000000000355

**Published:** 2023-12-22

**Authors:** Yiheng Zhang, Xu Liu, Shu Li, Chunlei Lin, Qian Ye, Yuying Wang, Juanli Wu, Yushuang Zhang, Han Gao, Tao Li, Yundong Qu, Yan Wang

**Affiliations:** 1 Department of Infectious Diseases and Hepatology, The Second Hospital of Shandong University, Jinan, China; 2 Department of Infectious Diseases, Weifang People’s Hospital, Weifang, China

## Abstract

**Background:**

Antiviral therapy improves the clinical outcomes of patients with HBV-related cirrhosis. In this study, we aimed to evaluate the incidence rate of HCC in patients with HBV-related recompensated, compensated, or decompensated cirrhosis based on the latest Baveno VII criteria.

**Methods:**

In this two-center retrospective study, HBV-related patients with cirrhosis were enrolled and treated with first-line nucleos(t)ide analogues therapy for at least 12 months. Participants were classified into 3 groups: (1) compensated group, (2) decompensated group, or (3) recompensated group according to Baveno VII criteria. Multivariate regression models and propensity score matching were used to identify the predictors of HCC.

**Results:**

Of the 404 patients recruited, during a median follow-up of 44.5 months (interquartile range 26.8, 57.0 months), 233 (57.7%), 100 (24.8%), and 71(17.6%) patients had compensated, recompensated, and decompensated cirrhosis. In total, 38 developed HCC. The cumulative incidence of HCC development at 2, 4, and 6 years was 1.3%, 5.4%, and 20.0% in the compensated group, 1.2%, 5.2%, and 24.5% in the recompensated group, and 2.1%, 23.6%, and 41.8% in the decompensated group, respectively. In the multivariate Cox regression model, compared with the recompensated group, the decompensated group had a significant increased risk for the development of HCC (aHR 2.55; 95% CI: 1.240–5.240; *p* = 0.027), while the compensated group had similar HCC risk for the development of HCC (aHR 1.41; 95% CI: 0.540–3.730; *p* = 0.835). Propensity score-matching analysis between the recompensated and compensated groups (84 pairs) and propensity score-matching analysis between the recompensated and decompensated groups (62 pairs) showed similar results.

**Conclusions:**

Achieving recompensation reduced the risk of HCC in patients with HBV-related decompensated cirrhosis, while the risk remained comparable to that of compensated cirrhosis.

## Graphical abstract

**Figure:**
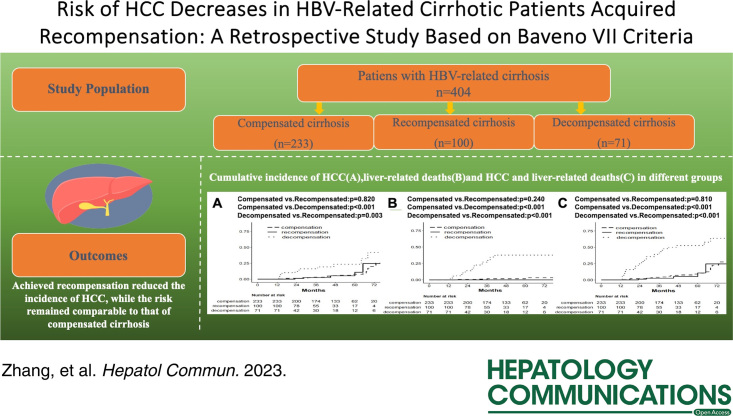

## INTRODUCTION

Chronic HBV infection is always an important health issue. According to World Health Organization figures, roughly 296 million individuals are living with chronic HBV infection, with about 1.5 million new cases of hepatitis B infection reported per year.^1^ Moreover, further analysis showed that HBV-related cirrhosis resulted in an estimated 331,000 deaths in 2019, and deaths from HBV-related liver cancer are estimated to be 192,000 in 2019, an increase from 156,000 in 2010.^2^ Patients with decompensated cirrhosis may have a poor prognosis and an increased risk of hospital readmission, development of HCC, liver transplantation, or death.

Fortunately, with the introduction of nucleos(t)ide analogues (NAs), some patients with hepatitis B-related decompensated cirrhosis may be able to avoid complications related to decompensation for an extended period of time. A 10-year follow-up study conducted in South Korea found that patients with decompensated hepatitis B cirrhosis experienced decreased Model for End-Stage Liver Disease (MELD) and Child-Pugh scores after receiving effective antiviral therapy, which is considered recompensation.^3^

The recompensation criteria and scoring system for forecasting recompensation in patients with HBV-related decompensated cirrhosis were thoroughly investigated in response to flaws identified in the 2 previous systems.^4,5^ The recent Baveno VII consensus, which advocated the novel hepatic recompensation criteria, proposes that the reversal of the structural and functional modifications of cirrhosis subsequent to the elimination or mitigation of the etiology of cirrhosis may partially decrease disease progression and attain an improved prognosis.^6^ There is strong evidence to suggest that patients meeting the Baveno VII criteria for recompensation, with stable improvement in liver function tests, can significantly reduce the incidence of complications, such as liver failure, ascites, variceal hemorrhage, and even death.^7,8^

However, few studies have focused on the impact of recompensation on the development of HCC. Therefore, we evaluated the risk of HCC according to the different stages of cirrhosis and compared the difference in the incidence rate of HCC between recompensated and compensated patients with cirrhosis.

## METHODS

### Study population

This is a retrospective cohort study of patients with HBV-related cirrhosis conducted between January 2016 and September 2023 at 2 participating hospitals: the Second Hospital of Shandong University and Weifang People’s Hospital. All patients initiated first-line potent NAs therapy, including entecavir, tenofovir disoproxil fumarate, and tenofovir alafenamide fumarate, within 6 months before enrollment or immediately after enrollment. This study received approval from the ethics committee at the Second Hospital of Shandong University. The inclusion criteria were as follows: (1) age ≥ 18 years old; (2) diagnosis of cirrhosis based on clinical, radiological, endoscopic, or histological findings. The exclusion criteria were as follows: (1) other concomitant chronic liver diseases, including hepatitis C, alcohol-associated liver disease, severe NAFLD, autoimmune liver disease, or drug-induced liver disease; (2) a previous history of HCC; (3) occurrence of HCC or death within 6 months after enrollment; and (4) follow-up duration less than 12 months. During the follow-up, patients were classified into 3 groups: the compensated group, recompensated group, and decompensated group. The recompensated group consisted of patients who had achieved recompensation according to Baveno VII criteria during follow-up. The decompensated group referred to patients who had experienced at least 1 decompensating event during the study period and did not achieve recompensation.

### Data collection

All data were collected from the electronic medical records. The baseline data was defined as the data collected at the initiation of antiviral therapy. Demographic characteristics included age and sex. The laboratory data included hemoglobin, platelet counts, alanine aminotransferase (ALT), aspartate aminotransferase, gamma-glutamy transpeptidase, total bilirubin, albumin, creatinine, serum sodium, alpha-fetoprotein, international normalized ratio, HBeAg status, serum HBV DNA (with a lower limit of detection of 20 IU/mL). The imaging data included ultrasonography, CT, and MRI. Clinical data included complications related to cirrhosis (ascites, HE, and esophageal variceal bleeding) and comorbidities (diabetes). The MELD and Child-Pugh scores were calculated using relevant data.

### Definitions

Liver cirrhosis was defined as the presence of any of the following: clinical features of portal hypertension; ultrasonography/CT/MRI findings associated with cirrhosis (surface nodularity, widening of fissures, enlargement of the lateral segments of the left lobe and caudate lobe, splenomegaly, collateral venous circulation, and an enlarged portal vein); endoscopic or histopathological evidence. Cirrhosis is classified as compensated or decompensated based on the absence or presence of related complications such as ascites, variceal bleeding, and/or HE. The definition of recompensation requires meeting all the following criteria: (1) sustained viral suppression for hepatitis B: undetectable serum HBV DNA (< 20 IU/ml) or HBsAg seroclearance; (2) resolution of ascites (discontinuation of diuretics) and HE (discontinuation of lactulose/rifaximin), and the absence of recurrent variceal bleeding for at least 12 months; and (3) stable improvement in liver function tests (MELD score < 10 and /or Child-Pugh Class A).^7^

### Study outcomes

The primary outcome was the occurrence of HCC. All patients underwent routine surveillance for HCC at least every 6 months with abdominal imaging and alpha-fetoprotein measurement. HCC was diagnosed on the basis of histological evidence or imaging criteria determined by dynamic CT and/or MRI (nodule > 1 cm, presence of arterial enhancement followed by portal/delayed-phase wash-out). Data were censored at the date of the last follow-up, the occurrence of HCC, or death, whichever occurred earliest.

### Statistics and analysis

Continuous variables were expressed as mean±SD or median (interquartile range), and one-way ANOVA or Kruskal-Wallis test was used for comparison between groups, as appropriate. Categorical variables were described as frequencies and percentages and compared using Pearson chi-squared test or Fisher exact test. The cumulative incidence of developing HCC was evaluated using the Kaplan-Meier method and compared using the log-rank test. The Cox proportional hazards model was performed to analyze the factors associated with HCC development. Significantly associated factors (*p* < 0.15) prefiltered in the univariate analyses were subsequently included in the multivariate Cox regression analysis using the backward stepwise method to identify independent prognostic factors. Propensity score matching (PSM) was performed to reduce the effect of selection bias and potential confounders between the study groups (compensated group vs. recompensated group; recompensated group vs. decompensated group). Then, the cumulative incidences of HCC between the study groups were compared after PSM. We also performed subgroup analysis by sex, age, HBeAg status, creatinine, Child-Pugh grade, and MELD score.All analyses were performed using R Statistical Software (Version 4.2.2, http://www.R-project.org, The R Foundation) and the Free Statistics analysis platform (Version 1.8, Beijing, China). Two-tailed *p* values < 0.05 were considered to be statistically significant.

## RESULTS

### Clinical characteristics of the study population

**FIGURE 1:**
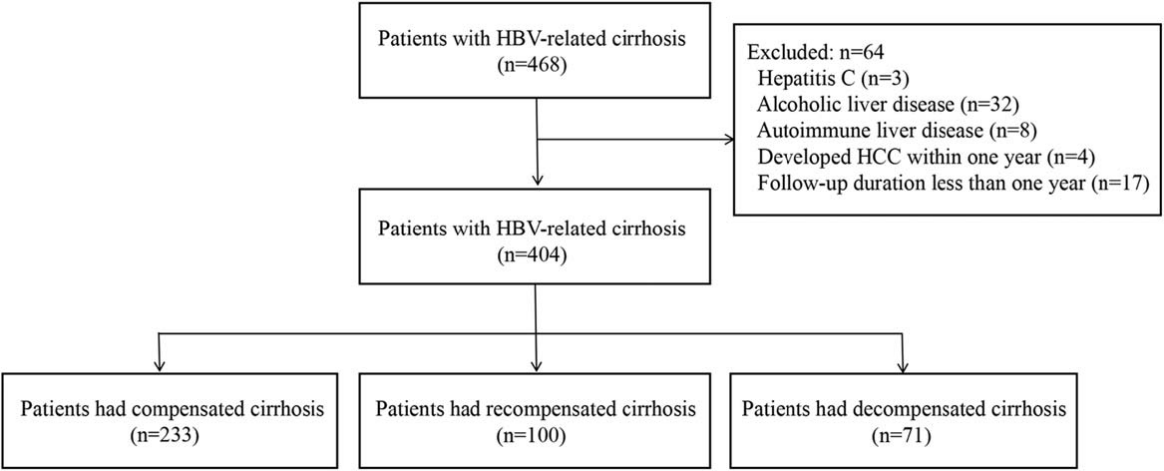
Flowchart of the study.

A total of 468 patients with HBV-related cirrhosis receiving first-line potent NAs were screened, and 404 were enrolled in the final cohort (Figure 1). Among these patients, 233 (57.7%) had compensated cirrhosis, 100 (24.8%) had recompensated cirrhosis, and 71(17.6%) had decompensated cirrhosis during the study period. At baseline, the mean age of the cohort was 51.3 years, and 66.8% were male. The mean Child-Pugh and MELD scores were 6.8 and 11.3, respectively. The decompensated events at baseline were ascites (144, 35.6%), HE (21, 5.2%), and esophageal variceal bleeding (27, 6.7%). Detailed baseline characteristics of the 3 groups are provided in Table 1.

**TABLE 1:** Baseline characteristics of the patients in the total cohort

Variables	All patients n = 404	Compensated cirrhosis n = 233	Recompensated cirrhosis n = 100	Decompensated cirrhosis n = 71	*p* ^a^	*p* ^b^	*p* ^c^	*p* ^d^
Male sex	270 (66.8)	166 (71.2)	52 (52)	52 (73.2)	0.001	<0.001	0.744	0.005
Follow-up duration, mo	44.5 (26.8, 57.0)	50.0 (35.0, 61.0)	39.0 (24.8, 53.2)	28.0 (16.5, 48.0)	<0.001	<0.001	<0.001	0.019
Age, y	51.3±11.3	49.1±10.8	52.8±11.0	56.3±11.2	<0.001	0.004	<0.001	0.043
Hemoglobin, g/L	128.2±30.9	141.7±24.8	110.9±28.9	108.2±28.8	<0.001	< 0.001	<0.001	0.539
Platelet, 10^9^/L	118.6±59.9	138.1±58.7	94.6±45.3	88.6±57.5	<0.001	<0.001	<0.001	0.443
ALT, U/L	46.5 (28.0, 113.5)	45.0 (26.0,132.0)	63.0 (34.8,148.8)	40.0 (27.5, 60.5)	0.006	0.044	0.104	<0.001
AST, U/L	50.0 (29.8, 107.0)	41.0 (25.0, 99.0)	77.0 (47.0,136.5)	53.0 (36.0, 85.0)	<0.001	<0.001	0.07	<0.001
GGT, U/L	44.5 (27.0, 86.2)	44.0 (27.0, 90.0)	51.0 (28.0, 86.2)	43.0 (28.0, 71.5)	0.708	0.466	0.9	0.449
Total bilirubin, umol/L	22.8 (14.6, 38.4)	17.9 (12.2, 26.7)	33.2 (20.9, 72.3)	30.9 (21.0, 44.6)	<0.001	<0.001	<0.001	0.288
Albumin, g/L	37.2±8.0	41.9±6.1	31.0±5.5	30.7±5.8	<0.001	<0.001	<0.001	0.668
Creatinine, mmol/L	65.5±17.8	67.4±14.1	61.1±20.3	65.7±23.6	0.014	0.001	0.476	0.175
NA^+^, mmol/L	139.7±3.5	140.7±2.2	138.4±4.7	138.3±4.1	<0.001	<0.001	<0.001	0.849
AFP, ng/ml	6.6 (3.0, 27.1)	4.8 (2.8, 13.0)	26.0 (8.8, 133.4)	5.1 (2.8, 14.0)	<0.001	<0.001	0.641	<0.001
INR	1.3±0.3	1.2±0.1	1.5±0.4	1.4±0.3	<0.001	<0.001	<0.001	0.08
HBV DNA, log_10_ IU/mL	5.0±2.1	4.9±2.2	5.6±1.9	4.6±2.1	0.003	0.003	0.421	0.002
HBeAg+,n (%)	185 (45.8)	110 (47.2)	54 (54)	21 (29.6)	0.005	0.256	0.009	0.002
Diabetes, n (%)	55 (13.6)	19 (8.2)	18 (18)	18 (25.4)	<0.001	0.009	<0.001	0.245
Child-Pugh score	6.8±2.1	5.4±0.8	8.7±2.0	8.5±1.6	<0.001	<0.001	<0.001	0.501
Child-Pugh stage (A/B/C)	54.7%/30.9% /14.4%	85.8%/13.7% /0.4%	14%/49% /37%	9.9%/62% /28.2%	<0.001	<0.001	<0.001	0.242
MELD score	11.3±4.4	9.3±2.9	14.5±4.9	13.3±4.3	<0.001	<0.001	<0.001	0.099
Decompensating events								
Ascites	144 (35.6)	/	84 (84)	60 (84.5)	0.001	/	/	0.929
HE	21 (5.2)	/	1 (14)	7 (9.9)	<0.001	/	/	0.416
Esophageal variceal bleeding	27 (6.7)	/	11 (11)	16 (22.5)	<0.001	/	/	0.042

a Chi-square or Fisher exact tests with Bonferroni correction compared all 3 groups of patients.

b Chi-square or Fisher exact tests with Bonferroni correction compared patients with compensated cirrhosis and patients with recompensation.

c Chi-square or Fisher exact tests with Bonferroni correction compared patients with compensated cirrhosis and patients with decompensation.

d Chi-square or Fisher exact tests with Bonferroni correction compared patients with decompensation and patients with recompensation.

Abbreviations: AFP, alpha-fetoprotein; ALB, albumin; ALT, alanine aminotransferase; AST, aspartate aminotransferase; GGT, γ-glutamyl transferase; INR, international normalized ratio; MELD, Model for End-Stage Liver Disease; PLT, platelet count.

### Risk of HCC between the recompensated versus decompensated or compensated groups

At a median (interquartile range) follow-up of 44.5 (26.8–57.0) months, 38 patients developed HCC, with 18 (7.7%) in the compensated group, 6 (6.0%) in the recompensated group, and 14 (19.7%) in the decompensated group. Additionally, 22 patients died of complications of cirrhosis, with 4 (1.7%) in the compensated group and 18 (25.4%) in the decompensated group. Furthermore, 1 patient died of lung cancer, and 1 patient died of traffic accident in the compensated group, while one patient underwent liver transplantation in the decompensated group.

**FIGURE 2:**
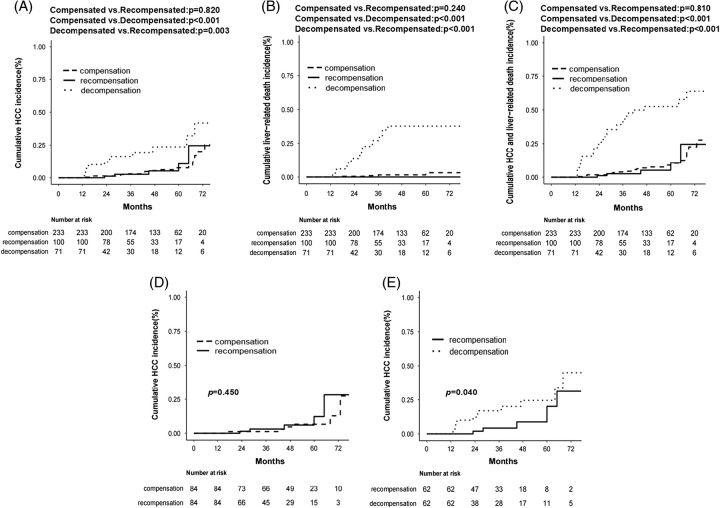
Cumulative incidence of HCC(A), liver-related deaths(B),and HCC and liver-related deaths(C) in different groups before PSM. Cumulative incidence of HCC in the compensated versus recompensated groups (D) and in the recompensated versus decompensated groups (E) after PSM. Abbreviation: PSM, propensity score matching.

The cumulative incidence of HCC development at 2, 4, and 6 years was 1.3%, 5.4%, and 20% in the compensated group, 1.2%, 5.2%, and 24.5% in the recompensated group, and 2.1%, 23.6%, and 41.8% in the decompensated group, respectively. There was a significant decrease in HCC incidence in the recompensated group compared to the decompensated group (*p* = 0.003, Figure 2A). However, there was no significant difference between the recompensated group and the compensated group (*p* = 0.820, Figure 2A). Furthermore, the beneficial effects of recompensation on survival and adverse events (HCC and liver-related deaths) were confirmed by Kaplan-Meier survival analysis (Figure 2B-C).

### Factors associated with HCC development

**FIGURE 3:**
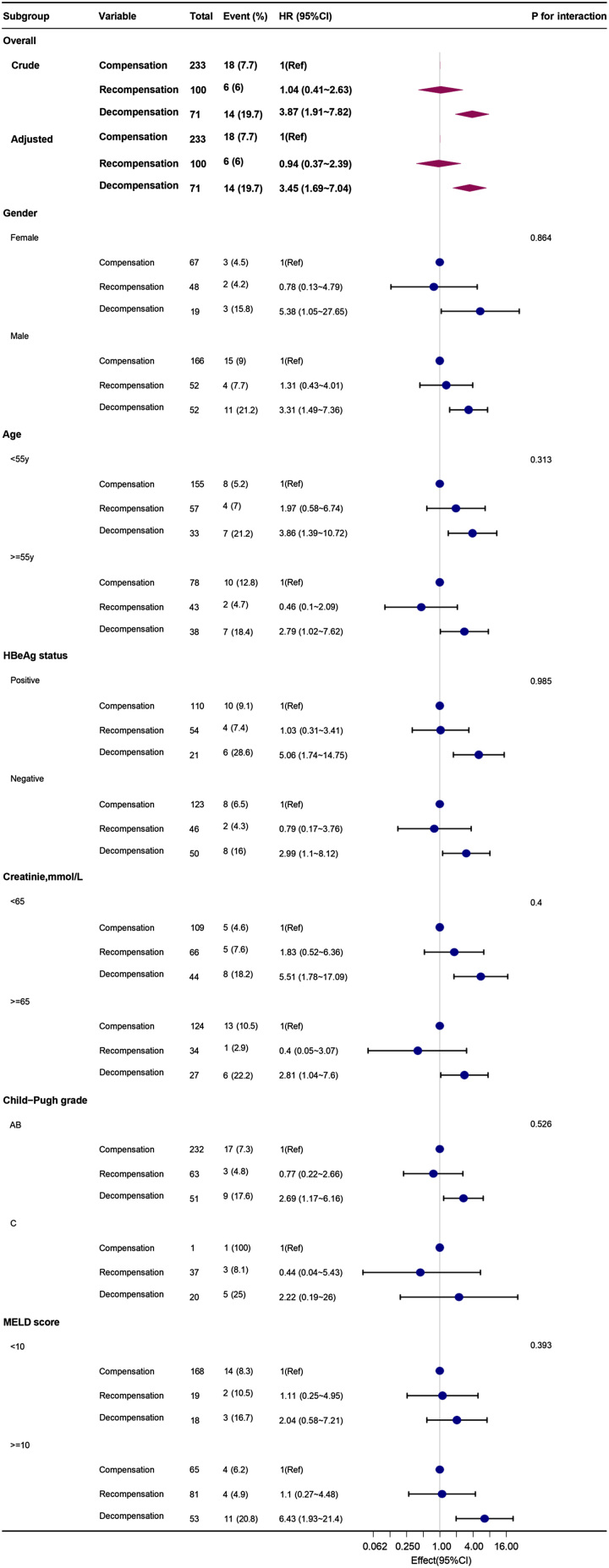
Subgroup analysis of incidence of HCC at baseline.

In the univariate Cox regression analysis, age and cirrhosis decompensation were significantly associated with HCC development. The multivariate Cox analysis further indicated that older age (aHR 1.04; 95% CI: 1.010–1.070; *p*=0.008) and cirrhosis decompensation (aHR 2.55; 95% CI: 1.240–5.240; *p*=0.027) were independent risk factors of HCC development (Table 2). Furthermore, we conducted subgroup analyses. The results showed that the effect of recompensation on HCC incidence was consistent across all 6 pre-specified subgroups (Figure 3).

**TABLE 2:** Analysis of factors linked to HCC at baseline

	Univariate analysis		Multivariate analysis	
Factors	HR (95% CI)	*p*	aHR (95% CI)	*p*
Male sex	1.870 (0.860, 4.080)	**0.116**	1.860 (0.760, 4.530)	0.078
Age	1.040 (1.010, 1.070)	**0.010**	1.040 (1.010, 1.070)	**0.008**
Recompensated cirrhosis	Referent	—	—	—
Compensated cirrhosis	1.040 (0.410, 2.630)	0.934	1.410 (0.540, 3.730)	0.835
Decompensated cirrhosis	3.870 (1.910, 7.820)	**0.007**	2.550 (1.240, 5.240)	**0.027**
Hemoglobin, g/L	1.002 (0.991, 1.014)	0.719	—	—
Platelet,10^9^/L	0.999 (0.994, 1.005)	0.810	—	—
ALT, U/L	1.000 (0.999, 1.001)	0.762	—	—
AST, U/L	1.000 (0.998, 1.002)	0.924	—	—
GGT, U/L	1.000 (0.996, 1.003)	0.773	—	—
TBIL, mmol/L	1.001(0.995, 1.008)	0.679	—	—
Albumin, g/L	0.990 (0.950, 1.030)	0.509	—	—
Creatinine, mmol/L	1.010 (1.000, 1.030)	**0.107**	1.010 (0.990, 1.020)	0.387
NA^+^, mmol/L	0.970 (0.900, 1.040)	0.374	—	—
AFP, ng/ml	0.998 (0.993, 1.002)	0.235	—	—
INR	1.25 (0.420, 3.660)	0.689	—	—
HBV DNA, log_10_ IU/mL	0.97 (0.830, 1.130)	0.698	—	—
Child-Pugh score	1.14 (0.990, 1.310)	**0.066**	1.110 (0.880, 1.400)	0.399
MELD score	1.04 (0.970, 1.110)	0.284	—	—

Note: the numbers in bold in Univariate analysis mean their statistical significance were lower than 0.15 or 0.05. The numbers in bold in Multivariate analysis mean their statistical significance were lower than 0.05.

Abbreviations: AFP, alpha-fetoprotein; ALB, albumin; ALT, alanine aminotransferase; AST, aspartate aminotransferase; GGT, γ-glutamyl transferase; INR, international normalized ratio; MELD, Model for End-Stage Liver Disease; PLT, platelet count; TBIL, total bilirubin.

### Risk of HCC after adjustment via PSM

We also performed PSM to compare the risk of HCC between the recompensated versus decompensated or compensated groups. Patients in the recompensated group,who were decompensated at baseline, achieved a significant improvement in liver function after 48 weeks of antiviral treatment.^7,9^ To ensure the comparability of variables between the recompensated group and the compensated group, we performed PSM using variables at 48 weeks of antiviral therapy, including sex, age, aspartate aminotransferase, total bilirubin, alpha-fetoprotein, Child-Pugh score, and MELD score (Table 3). Eighty-four pairs were successfully matched in the recompensated and compensated groups. The cumulative incidence of HCC development at 2, 4, and 6 years was 1.2%, 4.7%, and 12.8% in the compensated group and 1.4%, 6%, and 12.3% in the recompensated group, respectively. In the 84 PS-matched pairs, there was no significant statistical difference in the incidence of HCC between the 2 groups (*p* = 0.450, Figure 2D).

**TABLE 3:** Characteristics of compensated and recompensated cirrhosis patients before and after propensity score matching at treatment weeks 48

	Before PSM				After PSM			
Variables	Compensated cirrhosis n = 233 (70%)	Recompensated cirrhosis n = 100 (30%)	ASD	*p*	Compensated cirrhosis n = 84 (50%)	Recompensated cirrhosis n = 84(50%)	ASD	*p*
Male sex	166 (71.2)	52 (52.0)	0.404	<0.001	47 (56.0)	48 (57.1)	0.024	0.876
Age, y	49.1±10.8	52.8±11.0	0.343	0.004	52.6±10.6	51.6±11.1	0.092	0.55
AST, U/L	24.0 (20.0, 32.0)	29.0 (22.0, 39.2)	0.380	<0.001	27.5 (22.0, 36.8)	28.0 (22.0, 36.2)	0.013	0.973
Total bilirubin, mmol/L	14.9 (11.9, 21.7)	17.4 (12.6, 25.3)	0.316	0.022	20.4 (12.8, 26.2)	16.5 (11.9, 23.6)	0.161	0.131
AFP, ng/ml	2.9 (2.0, 4.1)	3.1 (2.2, 5.8)	0.125	0.048	3.3 (2.1, 4.5)	3.1 (2.2, 5.2)	0.107	0.913
Child-Pugh score	5.1±0.5	5.4±1.0	0.409	<0.001	5.2±0.6	5.3±0.7	0.093	0.547
MELD score	8.4±1.8	10.1±4.0	0.528	<0.001	9.3±2.1	9.3±2.4	0.046	0.825

Abbreviations: AFP, alpha-fetoprotein; AST, aspartate aminotransferase; MELD, Model for End-Stage Liver Disease; PSM, propensity score matching.

PSM was conducted between the recompensated group and the decompensated group at the baseline for sex, age, Cr, Child-Pugh score, and MELD score (Table 4). A total of 62 pairs were well-matched in the 2 groups. The cumulative incidence of HCC development at 2, 4, and 6 years was 1.9%, 8.8%, and 31.6% in the recompensated group and 12.2%, 24.5%, and 45% in the decompensated group, respectively. PSM analysis between the recompensated group and the decompensated group showed that the incidence of HCC in the recompensated group was significantly lower than that in the decompensated group (*p* = 0.040, Figure 2E).

**TABLE 4:** Baseline characteristics of recompensated and decompensated cirrhosis patients before and after propensity score matching

	Before PSM				After PSM			
Variables	Recompensated cirrhosis n = 100 (58.5%)	Decompensated cirrhosis n = 71(41.5%)	ASD	*p*	Recompensated cirrhosis n = 62 (50%)	Decompensated cirrhosis n = 62(50%)	ASD	*p*
Male sex	52 (52)	52 (73.2)	0.450	0.005	45 (72.6)	44 (71)	0.036	0.842
Age, y	52.8±11.0	56.3±11.2	0.316	0.043	54.6±11.3	55.9±10.6	0.115	0.524
Creatinine, mmol/L	61.1±20.3	65.7±23.6	0.209	0.175	63.2±19.7	64.5±23.9	0.057	0.656
Child-Pugh score	8.7±2.0	8.5±1.6	0.106	0.501	8.7±1.9	8.5±1.6	0.117	0.516
MELD score	14.5±4.9	13.3±4.3	0.260	0.099	13.3±4.2	13.5±4.5	0.052	0.773

Abbreviations: MELD, Model for End-Stage Liver Disease; PSM, propensity score matching.

### Factors associated with recompensation

During the follow-up period, 100 (58.5%) decompensated patients achieved hepatic recompensation. The univariate analysis showed several baseline variables associated with hepatic recompensation, including sex, age, and levels of ALT, aspartate aminotransferase, and HBV DNA. In the multivariate analysis, higher ALT level was independently associated with a higher likelihood of recompensation, while male sex and older age were significantly linked to a lower probability of recompensation (Table 5).

**TABLE 5:** Logistics analysis for prediction of recompensation at baseline

	Univariate analysis		Multivariate analysis	
Factors	HR (95% CI)	*p*	aHR (95% CI)	*p*
Male sex	0.40 (0.210, 0.760)	**0.006**	0.31 (0.150, 0.650)	**0.002**
Age	0.97 (0.940, 1.000)	**0.045**	0.97 (0.940, 1.000)	**0.046**
Hemoglobin, g/L	1.00 (1.000, 1.010)	0.443	—	—
Platelet, 10^9^/L	1.00 (0.990, 1.010)	0.537	—	—
ALT, U/L	1.01 (1.100, 1.020)	**0.003**	1.01 (1.000,1.010)	**0.022**
AST, U/L	1.01 (1.100, 1.010)	**0.020**	—	—
GGT, U/L	1.00 (1.000, 1.000)	0.764	—	—
Total bilirubin, mmol/L	1.00 (1.000, 1.010)	0.312	—	—
Albumin, g/L	1.01 (0.960, 1.070)	0.666	—	—
Creatinine, mmol/L	0.99 (0.980, 1.000)	0.182	—	—
Sodium, mmol/L	1.01 (0.940, 1.080)	0.848	—	—
AFP, ng/ml	1.00 (1.000, 1.000)	0.245	—	—
INR	2.33 (0.890, 6.080)	0.085	—	—
HBV DNA, log_10_ IU/mL	1.28 (1.100, 1.510)	**0.002**	1.17 (0.980, 1.400)	0.078

Note: the numbers in bold in Univariate analysis mean their statistical significance were lower than 0.15 or 0.05. The numbers in bold in Multivariate analysis mean their statistical significance were lower than 0.05

Abbreviations: AFP, alpha-fetoprotein; ALB, albumin; ALT, alanine aminotransferase; AST, aspartate aminotransferase; GGT, γ-glutamyl transferase; INR, international normalized ratio; MELD, Model for End-Stage Liver Disease; PLT, platelet count.

### Sensitivity analysis

To ensure the robustness of the findings, we performed a sensitivity analysis after excluding data from 52 patients from Weifang People’s Hospital. The multivariate Cox analysis showed that decompensated cirrhosis was associated with an ~threefold higher risk of HCC incidence (aHR 3.45; 95% CI: 1.180–10.090; *p* = 0.023) compared to recompensated cirrhosis, whereas recompensated and compensated cirrhosis had a comparable risk of HCC incidence (aHR 1.34; 95% CI: 0.330–5.390; *p*=0.681) (Supplemental Table S1, http://links.lww.com/HC9/A718, Supplemental Figure S1A, http://links.lww.com/HC9/A719). Next, we performed PSM to compare the risk of HCC between the recompensated versus decompensated or compensated groups, and we obtained similar results (Supplemental Tables S2–3, http://links.lww.com/HC9/A718, Supplemental Figure S1 B,C, http://links.lww.com/HC9/A719). These findings suggested that cirrhosis decompensation is a significant risk factor for HCC development while achieving recompensation decreases the risk even after excluding patients from a single center.

## DISCUSSION

Our current study revealed that achieving recompensation, compared to the decompensated group, significantly reduced the incidence of HCC. However, we found no statistically significant difference in HCC incidence between the recompensated and compensated groups. These results have significant implications for the management of HBV-related decompensated patients with cirrhosis with NAs, underscoring the importance of ongoing HCC surveillance even after recompensation.

To our knowledge, this is the first cohort study focused on HCC incidence after recompensation, according to Baveno VII definition^6^ in patients with HBV-related decompensated cirrhosis. For the first time, the Baveno VII consensus proposed an explicit definition and 3 requirements for recompensation. A recent prospective study further validated and defined the criteria for the stable improvement of liver function tests required by Baveno VII (MELD score < 10, and/or serum albumin, total bilirubin, and international normalized ratio within Child-Pugh A).^7^ However, despite the utility of the latest Baveno VII criteria in identifying recompensation patients, little is known about the long-term outcomes of such patients.^10^ A recent study from Hong Kong found that decompensated cirrhosis was associated with a higher risk of transplant-free survival compared to compensated cirrhosis, but there was no statistically significant difference in transplant-free survival between the recompensated and compensated groups in patients with HBV-related cirrhosis.^8^ Another study investigated the survival rate in patients with decompensated alcohol-associated cirrhosis after recompensation. The authors found that achieving recompensation resulted in a more than 90% risk reduction in liver-related mortality and a decreasing trend towards a lower HCC risk.^11^ Similarly, our study also demonstrated that achieving recompensation, as compared to the decompensated group, significantly reduced liver-related mortality, with comparable outcomes between the recompensated and compensated groups. For patients with HBV-related decompensated cirrhosis, the primary causes of liver-related mortality include liver failure and HCC. Antiviral therapy has been shown to be highly effective in reducing the incidence of liver failure by ameliorating necroinflammation and regressing fibrosis.^3,12^

However, the risk of HCC remains a significant concern. In our study, we paid special attention to this issue. Our research indicated that achieving recompensation through NAs therapy can significantly reduce the incidence of HCC in patients with HBV-related decompensated cirrhosis and eventually achieve a comparable HCC incidence to that of compensated patients with cirrhosis. Additionally, similar to previous studies, we found that older age is independently associated with a higher risk of HCC incidence.^13–16^ HCC from chronic hepatitis B infection is a result of the pro-oncogenic properties of the virus that can occur both directly ( integration of the virus genome in the host DNA) and indirectly because of rounds of liver cell necrosis and regeneration.^17–20^ HBV infection is often acquired perinatally in Asia; therefore, older age usually means long-standing infection and increases HCC risk by both the direct and indirect mechanisms. Our study suggested that achieving recompensation can attenuate but not completely eliminate the occurrence of HCC. Therefore, regular HCC screening remains imperative for patients who have achieved recompensation to ensure early detection and treatment, particularly for older individuals.

In our study, after antiviral therapy, 58.5% of decompensated patients (100/171) achieved recompensation according to Baveno VII criteria (MELD score <10, and/or serum albumin, total bilirubin, and international normalized ratio within Child-Pugh A). Our recompensation rate is similar to that reported in a multicenter, prospective study on HBV-related cirrhosis (56.2%, 159/283)^7^ but notably higher than that in a territory-wide study from Hong Kong (21.1%, 236/1116)^8^ and a study on alcohol-associated cirrhosis (18.1%, 37/204).^11^ This discrepancy may be attributed to variations in the baseline characteristics of the study populations and differences in the etiology of cirrhosis. Similar to previous reports, we found that high pre-treatment ALT level was independently associated with a higher recompensation rate on antiviral therapy.^5,7^

This suggested that patients with increased necro-inflammatory activity in the liver had better potential for response to antiviral therapy and subsequent hepatic recompensation.^21^ Similar to previous reports,^22^ we found that young age was associated with recompensation. Additionally, we also found that the female sex was independently associated with a higher recompensation rate, which may be attributed to the increased functionality of CD8+ T lymphocytes in the adaptive immune response to HBV infection in females.^23^

Our study has several limitations. First, this study was retrospectively designed, and selection bias may be a limitation. Nonetheless, we conducted propensity score-matching analysis and subgroup and sensitivity analyses, which consistently yielded similar results. Second, we did not assess the genotypes of HBV at baseline. Previous studies have shown that genotype C accounts for 85% of HBV genotypes in our region (northern China)^24^; therefore, we speculated that the differences in HCC risk resulting from genotype variations were not very noticeable. Third, we did not perform liver biopsies on patients who experienced recompensation. Future prospective studies may incorporate liver biopsies to confirm histological evidence of cirrhosis reversal, thereby deepening our understanding of recompensation.

**FIGURE 4:**
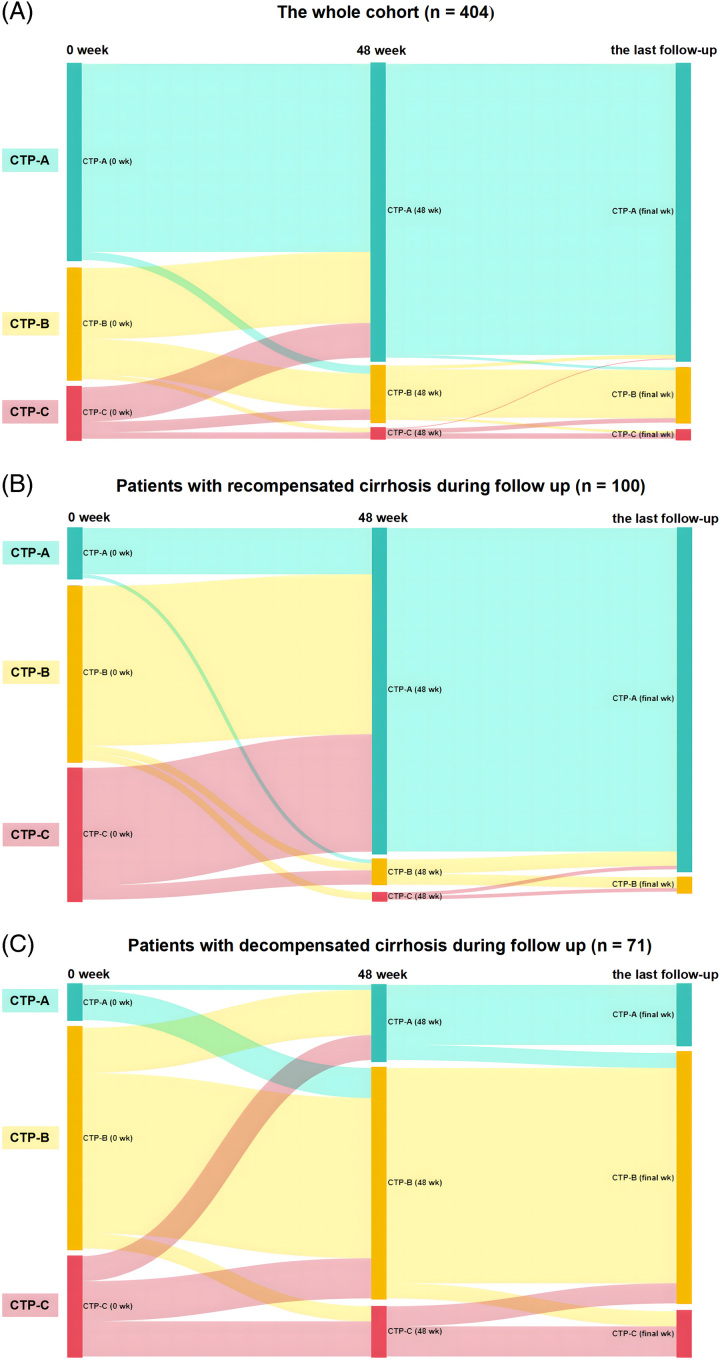
Sankey diagrams for the change of Child-Pugh scores from baseline to the last follow-up.(A) The whole cohort (n = 404). (B) Patients with clinical resolution of decompensating events (n = 100). (C) Patients without clinical resolution of decompensating events (n = 71).

In summary, our study indicated that achieving recompensation reduced the incidence of HCC in patients with HBV-related decompensated cirrhosis. Nevertheless, such patients remain at a comparable risk for HCC development compared to compensated patients with cirrhosis. Therefore, our findings support the early use of antiviral treatment to facilitate hepatic recompensation and highlight the importance of HCC screening in patients with HBV-related cirrhosis, even after recompensation (Figure 4).

## Supplementary Material

SUPPLEMENTARY MATERIAL
