# Supplementary material for: Risk of HCC decreases in HBV-related patients with cirrhosis acquired recompensation: A retrospective study based on Baveno VII criteria
Source: Hepatol Commun. 2023 Dec 22;8(1):e0355. doi: 10.1097/HC9.0000000000000355 (PMC10749709; doi:10.1097/HC9.0000000000000355)
Supplement: SUPPLEMENTARY MATERIAL [file hc9-8-e0355-s001.docx]

**Table S1. Analysis of factors linked to HCC at baseline.**

| **Factors** | **Univariate analysis** | |  | **Multivariate analysis** | |
| --- | --- | --- | --- | --- | --- |
|  | **HR(95%CI)** | ***p* value** |  | **aHR(95%CI)** | ***p* value** |
| Male sex | 1.890 (0.830,4.340) | **0.132** |  | 2.480 (1.030,5.980) | **0.043** |
| Age,years | 1.030 (1.000,1.060) | **0.040** |  | 1.040 (1.010,1.070) | **0.020** |
| Recompensated  cirrhosis |  |  |  |  | |
| Compensated  cirrhosis | 1.030 (0.380,2.800) | 0.955 |  | 1.340 (0.33,5.39) | 0.681 |
| Decompensated  cirrhosis | 3.950 (1.410,11.100) | **0.009** |  | 3.450 (1.18,10.09) | **0.023** |
| HGB, g/L | 1.006 (0.991,1.022) | 0.421 |  |  |  |
| PLT,10^9^ /L | 0.999 (0.994,1.005) | 0.752 |  |  |  |
| ALT, U/L | 1.000 (0.999,1.002) | 0.582 |  |  |  |
| AST, U/L | 1.000 (0.998,1.002) | 0.887 |  |  |  |
| GGT, U/L | 1.000(0.996,1.003) | 0.902 |  |  |  |
| TBIL, mmol/L | 1.002 (0.996,1.008) | 0.550 |  |  |  |
| ALB, g/L | 0.990 (0.950,1.030) | 0.529 |  |  |  |
| Creatinine, mmol/L | 1.010 (0.990,1.030) | 0.197 |  |  |  |
| NA^+^, mmol/L | 0.960 (0.900,1.030) | 0.252 |  |  |  |
| AFP,ng/ml | 1.000 (0.994,1.002) | 0.273 |  |  |  |
| INR | 1.390 (0.480,4.020) | 0.548 |  |  |  |
| HBV DNA,  log_10_ IU/mL | 0.980 (0.840,1.140) | 0.770 |  |  |  |
| Child–Pugh score | 1.150 (1,100,1.330) | **0.056** |  | 1.13 (0.89~1.44) | 0.323 |
| MELD score | 1.050 (0.980,1.120) | 0.200 |  |  |  |

ALT, alanine aminotransferase;AST,aspartate aminotransferase;GGT,γ-glutamyl transferase; PLT, platelet count;MELD, model for end-stage liver disease; AFP,alpha-fetoprotein;ALB, albumin; HGB, haemoglobin; INR, international normalised ratio; TBIL, total bilirubin;

**Table S2. Characteristics of compensated and recompensated cirrhosis patients before and after propensity score matching at treatment weeks 48.**

| **Variables** | **Before PSM** | | | |  | **After PSM** | | | |
| --- | --- | --- | --- | --- | --- | --- | --- | --- | --- |
|  | **Compensated**  **Cirrhosis**  **n =203(70.7%)** | **Recompensated**  **Cirrhosis**  **n =84（29.3%)** | **ASD** | ***p* value** |  | **Compensated**  **Cirrhosis**  **n =74 (50%)** | **Recompensated**  **Cirrhosis**  **n = 74(50%)** | **ASD** | ***p* value** |
| Male sex | 46 (54.8) | 147 (72.4) | 0.373 | 0.004 |  | 41 (55.4) | 40 (54.1) | 0.027 | 0.869 |
| Age,years | 52.7 ± 11.8 | 49.1 ± 10.7 | 0.32 | 0.012 |  | 51.8 ± 11.6 | 52.2 ± 10.5 | 0.042 | 0.8 |
| AST, U/L | 28.5 (22.0, 38.2) | 24.0 (20.0, 32.0) | 0.348 | 0.005 |  | 27.0 (22.0, 35.0) | 29.5 (22.0, 39.8) | 0.083 | 0.325 |
| TBIL, mmol/L | 16.8 (11.9, 24.4) | 14.3 (11.5, 20.9) | 0.31 | 0.038 |  | 16.2 (11.8, 22.4) | 17.4 (12.9, 23.9) | 0.099 | 0.421 |
| AFP,ng/ml | 3.3 (2.2, 6.5) | 2.9 (1.8, 4.1) | 0.382 | 0.024 |  | 3.0 (2.1, 5.2) | 3.4 (2.2, 7.0) | 0.06 | 0.269 |
| Child-Pugh score | 5.4 ± 1.0 | 5.1 ± 0.5 | 0.498 | < 0.001 |  | 5.3 ± 0.6 | 5.3 ± 0.8 | 0.054 | 0.713 |
| MELD score | 9.9 ± 4.2 | 8.3 ± 1.8 | 0.373 | < 0.001 |  | 9.0 ± 2.4 | 8.9 ± 2.1 | 0.027 | 0.77 |

AST,aspartate aminotransferase;MELD, model for end-stage liver disease; AFP,alpha-fetoprotein;

TBIL, total bilirubin;

**Table S3. Baseline characteristics of Recompensated and Decompensated cirrhosis patients before and after propensity score matching.**

| **Variables** | **Before PSM** | | | |  | **After PSM** | | | |
| --- | --- | --- | --- | --- | --- | --- | --- | --- | --- |
|  | **Recompensated**  **Cirrhosis**  **n =84 (56.4%)** | **Decompensated**  **Cirrhosis**  **n =65(43.6%)** | **ASD** | ***p* value** |  | **Recompensated**  **Cirrhosis**  **n =52 (50%)** | **Decompensated**  **Cirrhosis**  **n = 52(50%)** | **ASD** | ***p* value** |
| Male sex | 46 (54.8) | 48 (73.8) | 0.406 | 0.002 |  | 40 (76.9) | 37 (71.2) | 0.132 | 0.502 |
| Age,years | 52.7 ± 11.8 | 57.0 ± 10.9 | 0.381 | 0.023 |  | 54.5 ± 10.5 | 55.8 ± 10.7 | 0.116 | 0.555 |
| Child-Pugh score | 8.7 ± 2.0 | 8.6 ± 1.7 | 0.076 | 0.648 |  | 8.8 ± 2.0 | 8.6 ± 1.7 | 0.126 | 0.522 |
| MELD score | 14.5 ± 5.2 | 13.5 ± 4.3 | 0.217 | 0.174 |  | 14.0 ± 4.6 | 13.5 ± 4.6 | 0.111 | 0.538 |

MELD, model for end-stage liver disease;
