# Supplementary figures and images for: Risk of HCC decreases in HBV-related patients with cirrhosis acquired recompensation: A retrospective study based on Baveno VII criteria
Source: Hepatol Commun. 2023 Dec 22;8(1):e0355. doi: 10.1097/HC9.0000000000000355 (PMC10749709; doi:10.1097/HC9.0000000000000355)

## Slide 1
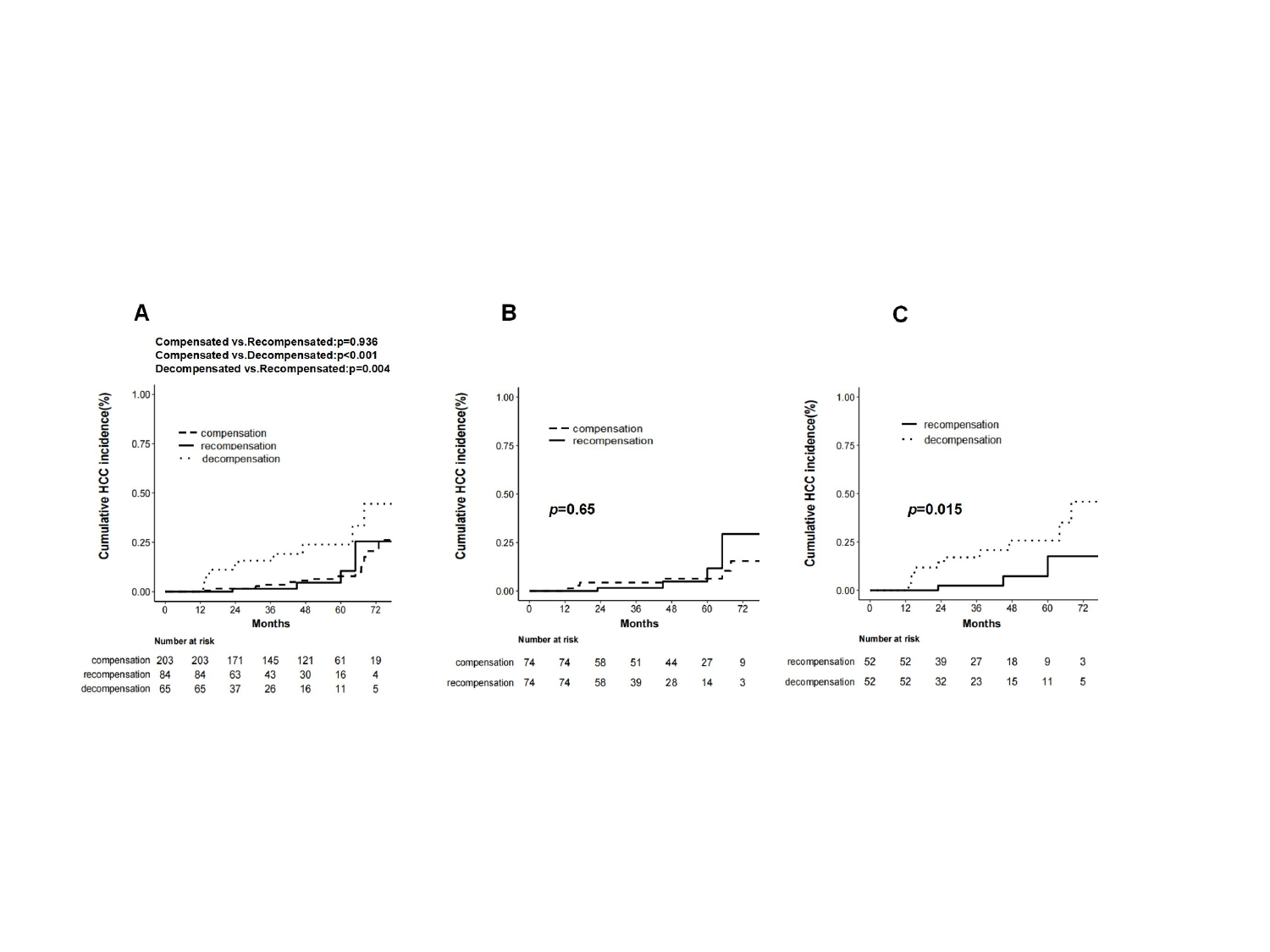

Supplement: SUPPLEMENTARY MATERIAL [file hc9-8-e0355-s002.ppt]
